# Predictors of 1-Year Perceived Recovery, Absenteeism, and Expenses Due to Low Back Pain in Workers Receiving Mechanical Diagnosis and Therapy: A Prospective Cohort Study

**DOI:** 10.3390/healthcare11091293

**Published:** 2023-04-30

**Authors:** Hiroshi Takasaki

**Affiliations:** Department of Physical Therapy, Saitama Prefectural University, Koshigaya 343-8540, Japan; physical.therapy.takasaki@gmail.com; Tel.: +81-048-973-4706

**Keywords:** absenteeism, health expenditures, therapeutic alliance, treatment adherence and compliance, self-management

## Abstract

This multicenter prospective cohort study aimed to preliminarily explore statistically relevant modifiable and predetermined factors for 1-year perceived recovery, absenteeism, and personal expenses in workers who received Mechanical Diagnosis and Therapy (MDT) for low back pain (LBP). Three stepwise multiple regression models were explored with 42 independent variables, including (1) socio-demographic factors; (2) risk stratification; (3) pain-related variables, psychological variables, and behavioral variables at baseline and changes after a month; (4) therapeutic alliance and exercise adherence at 1-month follow-up; and (5) MDT classification and therapist levels. Data from 58 participants were analyzed, after which a model with a medium effect size was developed for 1-year perceived recovery only. Consequently, patients with derangement syndrome were expected to have improved 1-year perceived recovery, with expected predetermined prognostic factors including shorter symptom duration, self-management skills to lead a healthy life, and less pain catastrophization at baseline. A stronger therapeutic alliance between patient and therapist during the 1-month MDT intervention was identified as an expected modifiable prognostic factor. It may be difficult to accurately predict the annual absenteeism and personal expenses due to LBP given the weak to low effect sizes of the developed models.

## 1. Introduction

Among musculoskeletal disorders, low back pain (LBP) remains the leading cause of absenteeism [1,2], with a significant increasing impact on both individuals and the wider socio-economy [3,4]. Before the coronavirus disease 2019 (COVID-19) pandemic, statistical data from the UK in 2019 showed that 28.4 million days were lost due to sickness absences related to LBP and neck pain, the second most common (20.6%) reason for absenteeism after minor illness [5]. Besides the impact of absenteeism due to LBP on productivity, personal expenses are significant [3] due to direct medical costs, such as treatment and medication, and indirect costs, such as bedding renewal fees and complementary therapy fees.

The McKenzie method of Mechanical Diagnosis and Therapy (MDT) has been reported to be one of the most common approaches for LBP management in Ireland, the United States, and the United Kingdom [6,7,8,9]. With the use of MDT, there has been increasing preliminary evidence for its cost savings and health utilization for LBP [10,11,12,13]. MDT is a conservative management system for musculoskeletal disorders. It is a patient-centered and biopsychosocial approach with risk management according to certain MDT subgroup classifications. Tsuge et al. [14] reported that the subgroup classification of derangement syndrome was a favorable prognostic factor for days and number of sessions needed until discharge among those who received MDT. Risk stratification using the STarT Back Screening Tool was also a favorable prognostic factor for the number of sessions needed until discharge [14]. However, this study was a retrospective chart review study that did not comprehensively include potential prognostic factors, and the developed models had limited power (*R*^2^ = 0.17–0.19). Thus, factors that contribute to a favorable prognosis in response to MDT still remain obscure.

Previous studies have shown that MDT interventions not only improve physical functioning and pain-related variables, including the degree of pain and disability [15,16], but also improve psychological status, including pain self-efficacy, kinesiophobia, and pain catastrophization [17,18], and promote behavioral changes toward self-management [19]. Furthermore, MDT can establish a strong patient–therapist alliance [20] and facilitate excellent exercise adherence [15]. By developing a predictive model for perceived recovery in one year, annual number of days off work, and personal expenses that comprehensively incorporates factors that can be modified by MDT interventions within a short time as well as predetermined factors (e.g., demographics and initial medical condition), we can identify areas needing focus in our current management of patients to improve future outcomes through MDT interventions.

Thus, this study aimed to preliminarily explore statistically relevant modifiable and predetermined factors at the initial MDT session and at 1-month follow-up for (1) perceived recovery after one year, (2) annual days off work, and (3) personal expenses for one year in workers with LBP.

## 2. Materials and Methods

### 2.1. Design

This was a multicenter 1-year prospective cohort study. A multiple regression model was developed using dependent variables collected at 1-year follow-up, such as 11-point Perceived Recovery of Change Scale (PRCS), self-reported annual days off work due to LBP, and self-reported annual personal expenses due to LBP, and independent variables collected at the initial MDT session and at 1-month follow-up, such as pain-related, psychological, and behavioral variables. Data were collected via mail or web survey, depending on the participants’ preference.

### 2.2. Participants

Participants were recruited from 12 hospitals and clinics between November 2019 and December 2020. The inclusion criteria were as follows: (1) full-time workers, including those who are self-employed, who had received MDT by certified MDT practitioners with a primary complaint of LBP, (2) those aged 18–60 years, and (3) those who had received MDT for LBP or LBP-related lower extremity symptoms. The exclusion criteria were as follows: (1) those who were hospitalized for reasons other than LBP during the study period and (2) those who had a differential diagnosis of non-musculoskeletal disorders for their LBP, such as cancer, during the study period.

### 2.3. Therapists

Interventions were given by 23 registered MDT practitioners who had obtained their credentials (n = 18) or diploma (n = 5) from 13 medical institutions across Japan. In this study, the MDT practitioners were blinded to their patients’ data until the final survey one year after the initial MDT session.

### 2.4. Interventions

Interventions were based on MDT [21] and individualized for each participant. The treatment schedule and discharge timeline were also decided between the therapists and participants.

MDT is a patient-centered approach that promotes active patient participation in treatment decision-making, corrects inappropriate patient perceptions regarding pain, and enhances patient self-management skills through assessment and intervention based on a biopsychosocial perspective. MDT determines the treatment strategy based on 13 possible classifications for LBP: derangement syndrome, dysfunction syndrome, posture syndrome, adherent nerve root, serious pathologies, chronic pain syndrome, inflammatory, mechanically inconclusive, mechanically unresponsive radiculopathy, post-surgery, sacroiliac joint/pregnancy-related pelvic girdle pain, spinal stenosis, and trauma/recovering trauma. The load used is adjusted according to the characteristics of each MDT classification and the stage of recovery to obtain maximum functional recovery with minimum risk. For example, in the major classification of derangement syndrome, exercise therapy, manual therapy, postural education, and home exercises are prescribed according to directional preference, which is a direction of mechanical loading that results in instant and long-lasting symptom reduction. No rules have been established for specific hands-on techniques or exercise methods and capacities; however, feasible and effective interventions are prescribed through discussion with the patient. After symptoms disappear, physical function should be improved through exercise therapy to prevent recurrence if inadequate physical function is present. For instance, cases with mechanically unresponsive radiculopathy can undergo another mechanical assessment after receiving transforaminal epidural steroid injections to explore the possibility of changes to derangement syndrome [22]. If the classification of mechanically unresponsive radiculopathy remains unchanged, the patient should be provided with information on the advantages and disadvantages of conservative therapy and surgery as well as prognosis, with the therapist undertaking shared decision-making regarding the future management plan. MDT-certified practitioners exhibit excellent intra-examiner reliability of the MDT classifications [23]; thus, a change in therapist is not expected to make a substantive difference in treatment within the same MDT certification level. 

### 2.5. Outcome Measures

#### 2.5.1. Primary Outcomes

The dependent variables included (1) an 11-point PRCS (−5 [very much worse] to 5 [completely recovered]) [24] collected at 12 months after the initial MDT session; (2) self-reported annual days off work due to LBP, where a day with telework was counted as a working day; and (3) self-reported annual personal expenses due to LBP in yen (JPY), including direct medical costs, such as treatment and medication for LBP, and indirect costs, such as bedding renewal fees and complementary therapy fees. The latter two measures were collected at 1, 3, 6, and 12 months after the initial MDT session, and the summed values were used in regression modeling.

#### 2.5.2. Secondary Outcomes

In brief, independent variables included (1) socio-demographic factors; (2) risk stratification; (3) pain-related, psychological, and behavioral variables at baseline and changes after a month; (4) therapeutic alliance and exercise adherence at 1-month follow-up; and (5) MDT classification and therapist levels. All of these variables were expected to be associated with MDT [14,15,16,17,18,19,20,25,26]. In this study, each patient-reported outcome measure that had a Japanese version and established reliability and/or validity was selected. Among the 42 independent variables included in the regression model, data for 39 variables were collected through a survey, whereas data for the other three variables (the 12-item Örebro musculoskeletal screening questionnaire [27,28] [ÖMSQ-12-J], MDT classification, and therapist levels) were collected from the therapists at the end of the 1-year follow-up of the participants. 

The following socio-demographic factors assessed at the initial MDT session were included in the regression model: (1) age, (2) gender, (3) body mass index calculated using self-reported height and weight, (4) job satisfaction [29,30] (“are you satisfied with your current job?” satisfaction category [yes and relatively yes] or dissatisfaction category [relatively no and no]), (5) history of recurrent LBP [31] (less than three times or not) [32], (6) job and physical activities in a typical workday (the two categories of physically demanding or not physically demanding work [29,33] were repeatedly classified by four research collaborators until a consensus was reached), and (7) symptom duration of the current episode [31] before the initial MDT session (less than three months or not). 

The following two risk stratification variables assessed at the initial MDT session were included in the regression model: the ÖMSQ-12-J (0–120, a higher total score indicating greater risk for poor prognosis) and the STarT Back Screening Tool [34] (low-risk group [total score < 4] or medium-high-risk group [total score ≥ 4] [14]). These two measures are similar but differ in their data acquisition methods. Given that the STarT Back Screening Tool was collected merely for research purposes, patients who participated in the study responded with a survey package. On the other hand, the ÖMSQ-12-J was expected to be utilized by MDT therapists to support yellow flag assessments [28]. Moreover, given that unskilled therapists might overlook a patient’s psychological problems in the absence of a screening tool [18,35], ÖMSQ-12-J was completed at the initial visit as a part of the examination and used for clinical reasoning. 

The following pain-related variables were included in the regression model: (1) symptom location [15] (0–6, with greater scores indicating more distal pain), (2) pain intensity according to the four-item Pain Intensity Measure [36] (0–40, with higher total scores indicating greater pain intensity), (3) disability according to the Oswestry Disability Index [37,38] (ODI) (with higher % scores indicating greater disability), and (4) symptoms indicating central sensitization according to the Central Sensitization Inventory [39,40] (0–40, with higher total scores indicating greater magnitude of symptoms indicating central sensitization). The following psychological variables were included in the regression model: (1) confidence in being able to do a particular behavior or task despite pain according to the Pain Self-Efficacy Questionnaire (PSEQ) [41,42] (0–60, with higher total scores indicating greater confidence in being able to do a particular behavior or task despite pain), (2) kinesiophobia according to the Tampa Scale for Kinesiophobia [43,44] (17–68, with higher total scores indicating greater kinesiophobia), (3) pain catastrophization according to the Pain Catastrophization Scale [45,46] (0–52, with higher total scores indicating greater pain catastrophization). The following behavioral variables were included in the regression model: attitude and skills for self-management according to the Health Education Impact Questionnaire (HeiQ) [47,48], which is composed of eight psychometric properties, including self-monitoring and insight, skill and technique acquisition, health-directed behavior, positive and active engagement in life, emotional distress, constructive attitudes and approaches, social integration and support, and health services navigation (1–4, with greater mean scores indicating more positive attitude and skill acquisition toward self-management). In HeiQ, since the cut-off value for favorable changes in each psychometric property is known, data were categorized according to whether there was an improvement over the cut-off value before or after the 1-month MDT and incorporated into the regression modeling. Additionally, PRCS (≥2 indicating improvement [24]) assessed at the 1-month follow-up were also included in the regression modeling. 

For therapeutic alliance, the five-item Modified Working Alliance Inventory-Short Form Client Japanese musculoskeletal version [49] (0–5, with higher total scores indicating greater working alliance) was included in the regression model. For exercise adherence, the Japanese version of the Exercise Adherence Rating Scale (EARS-J) [50] (with greater % scores indicating greater exercise adherence) was included in the regression model. 

For MDT classification, an MDT classification out of the 13 classifications for each participant was obtained from each MDT therapist after the data collection period. The MDT classifications were converted into a binary scale reflecting the absence or presence of derangement syndrome based on a possible prognostic factor identified in a previous study [14]. For therapist levels, the variable was converted into a binary scale (credential MDT or diploma MDT) and included in the regression model.

### 2.6. Data Analysis

Data analysis was performed using IBM SPSS Statistics for Windows, version 28.0 (Armonk, NY, USA: IBM Corp). Stepwise multiple regression analysis was performed using the Alpha-to-Enter significance level at 5% and the Alpha-to-Remove significance level at 10%. Statistical significance was set at 5%. The *R*^2^ value was interpreted as follows: <0.3, a none to very weak effect size; 0.3–0.5, a weak to low effect size; 0.5–0.7, a moderate effect size; and >0.7, a strong effect size [51]. The characteristics of participants were summarized using descriptive statistics. 

Given the exploratory nature of this study, no rigorous sample size estimation was performed. Owing to the study duration and budget constraints, an attempt was made to collect data from 170 participants.

## 3. Results

### 3.1. Participants

Participant recruitment was conducted from November 2019 to December 2020, but only a total of 68 patients participated in the initial survey. Of these sixty-eight participants, one was diagnosed with cancer and three withdrew consent at the 1-month follow-up, all of whom were excluded from data analysis. Consequently, data for 64 participants were included (32 men and 32 women, with a mean for age of 45.1 [SD 8.5] years). Data for six participants were excluded from multiple regression analysis due to the lack of follow-up data at 1 or 12 months, resulting in an analysis including 58 patients whose demographic characteristics are summarized in Table 1. Data imputation was not undertaken. At the 1-month follow-up, 50 of the 60 analyzed participants (83%) reported improvement, with the mean PRCS being 2.6 (SD 1.3) among the 58 participants who were included in the multiple regression analysis. The secondary outcomes at the baseline and 1-month follow-up are summarized in Table 1 and Table 2. At the 12-month follow-up, 57 of the 61 analyzed participants (93%) reported improvement, whereas 9.8% (6/61) reported complete recovery, with the mean PRCS being 3.4 (SD 1.3) among the 58 participants who were included in the multiple regression analysis. The mean values for the self-reported annual days off work due to LBP and self-reported annual personal expenses due to LBP were 2.0 days (SD 2.9 days) and JPY 67,347.2 (SD JPY 132,478.3), respectively, among the 58 participants who were included in the multiple regression analysis. Among the included participants, twenty were from four institutions in Tokyo; twenty were from an institution in Niigata Prefecture; nine were from an institution in Saga Prefecture; six were from an institution in Saitama Prefecture; two each were from an institution in Hokkaido, Yamaguchi, and Wakayama Prefectures; and one each was from an institution in Miyazaki, Hyogo, and Aichi Prefectures. 

### 3.2. Perceived Recovery of Change Scale

For the PRCS after one year, there were five statistically significant contributing factors, including one modifiable factor of therapeutic alliance (Table 3). The *R*^2^ values indicated a moderate effect size. The Durbin–Watson statistic was 2.22. There was one outlier for which the predicted value of the measured value was above 3 SD.

### 3.3. Annual Days off Work for One Year

For annual days off work for one year, only the PSEQ scores at the initial MDT session were a statistically significant contributing factor (Table 4). The *R*^2^ values indicated a none–very weak effect size. The Durbin–Watson statistic was 0.99. There were three outliers for which the predicted value of the measured value was above 3 SD.

### 3.4. Personal Expenses for One Year

For personal expenses for one year, there were four statistically significant contributing factors, including two modifiable factors of exercise adherence and disability (Table 5). The *R*^2^ values indicated a weak–low effect size. The Durbin–Watson statistic was 1.91. There were two outliers for which the predicted value of the measured value was above 3 SD.

## 4. Discussion

To the best of the author’s knowledge, this is the first study to develop predictive models of not only the subjective improvement after one year but also of the annual number of days off work and personal expenses for one year for those who received MDT. At least one statistically significant contributing factor was detected for each model, which would be rich in suggestions for the emergence of further research to fully understand the features of MDT and what should be done with particular awareness during the MDT in one month. 

A model with a medium effect size was created for the PRCS one year after MDT initiation. This result can be interpreted as containing clinical implications for predicting subjective improvement at one year. The included factors were the MDT classification of derangement syndrome; symptom duration; the skill and technique acquisition scores in the HeiQ and Pain Catastrophization Scale at baseline; and therapeutic alliance at 1-month follow-up. The findings of this MDT classification as an important predictor are consistent with those of previous studies [14,52]. It is not surprising that long history and high pain catastrophization are relevant to poor prognosis [53,54], whereas it is interesting that the skill and technique acquisition score in the HeiQ at baseline is a contributing factor. The high score at the time of the first visit may indicate that the patients have a strong attitude toward internal health locus of control and have tried self-management in their own way. In other words, it is important for such patients to have successful experiences of self-management through MDT, rather than excessive hands-on techniques that may lead to dependence on the therapist. More importantly, the construction of the therapeutic alliance within the first month of treatment was a modifiable factor in prognosis. This finding accords with previous findings that the therapeutic alliance affects the effectiveness and satisfaction of the treatment [55,56]. Therefore, further research is needed on how to enhance the therapeutic alliance, and it may be important to strengthen communication skills to increase patient autonomy [57]. In fact, the preliminary research findings show that this communication ability is not patient-dependent, but therapist-dependent, and those with MDT diploma training have higher levels of this communication ability than those with MDT credentials or therapists who have not studied MDT [58].

Only the PSEQ score at the initial MDT session was a statistically significant contributing factor for the annual days off work for one year. However, the effect size of this model was none to very weak and of limited clinical significance. This result indicates that outcomes other than those sampled in this study may be important predictors. Although the following factors may be considered as predictors, they may not be modifiable factors. Moreover, even if they are modifiable factors, future research is required to determine whether MDT can modify these factors: presentism [59]; work ability and feelings of depersonalization or emotional distance from work (burnout) [60]; pathological mental disorders [61]; and social factors [62], such as previous sick leave and unemployment; socio-economic status; return-to-work coordination; work environment; and peer support. 

A model with a weak to low effect size was created for the personal expenses due to LBP for one year. This model may provide clinical implications to reduce personal expenses due to LBP for one year. The included factors were the health-directed behavior scores in the HeiQ, ÖMSQ-12-J scores at baseline, EARS-J scores at 1-month follow-up, and reduction of ODI scores through one month. The contributing factors of the ÖMSQ-12-J scores at baseline, which is a screening tool for poor prognosis, and the reduction of ODI scores through one month, which is a modifiable factor, may be associated. These factors would indicate the importance of both the therapist and the patient to work together toward the same goal of disability reduction during the first month of treatment. Regarding behaviors for exercises, there were interesting findings: (1) those who scored high in health-directed behavior scores in the pre-MDT in the HeiQ, that is, those who had high levels of healthful behaviors including prevention, diet, and exercise to begin with, saved expenses as a result, and (2) those who scored high in the EARS-J, that is, those who had high levels of exercise adherence at 1-month follow-up, had high personal expenses. Helping patients acquire individualized self-management skills is an important aspect of LBP managements [63,64,65] and is emphasized in MDT [21]. Similar to a type of behavior change through LBP managements [66,67], people who originally had little commitment to self-management have realized the importance of self-management through MDT and increased their adherence to exercise, and might have made new investments in creating a suitable environment and in body building, resulting in higher expenses over the year. 

This study had some limitations. First, the data collection period was inadvertently affected by the COVID-19 pandemic. Owing to the suspension of medical institutions, the number of data samples from each institution was smaller than originally expected. However, a stepwise method was used to generate models for only up to five variables in this study, satisfying the empirical rule on requiring a sample of 10 persons for each variable during multiple regression analysis. Based on the statistically relevant factors identified in this study, future studies are necessary to develop a theoretical model that includes factors clinically relevant for predicting the prognosis of MDT and to test the fit of the model using a forced entry method with a sufficient sample size. Second, it is possible that patients who would have received treatment at a medical institution prior to COVID-19 did not seek medical care for the fear of contracting COVID-19. The COVID-19 pandemic may also have promoted differences in the recovery process, number of days off work, and personal expenses due to LBP due to the change in work style to telecommuting and changes in daily lifestyle. Therefore, caution should be taken while comparing the results of this study with situations after the COVID-19 convergence or before the COVID-19 pandemic. Third, data on the number of days off work and personal expenses were collected through self-reporting; thus, the information may be less reliable than that obtained using more robust data collection methods, such as the use of receipt data and company attendance records. However, given that the study results were analyzed after the data collection was completed, there is no possibility that each participant’s prior knowledge of the results of this study caused any bias that could have affected the self-reported data. Fourth, because this study did not control for interventions, it is not possible to rule out the influence of differences in treatment received on the outcome at the 12-month follow-up, which is a limitation of prospective cohort studies that include treatment. However, MDT-certified practitioners exhibit excellent intra-examiner reliability of MDT classification [23]. Considering that MDT determines the treatment strategy based on MDT classification identified through interventions, less variations in treatment are expected compared with other treatment approaches. 

In conclusion, among workers whose LBP was treated with MDT, those with an MDT classification of derangement syndrome can be expected to have better perceived recovery after one year. Expected predetermined prognostic factors included shorter symptom duration, self-management skills to lead a healthy life, and less pain catastrophization at baseline. Moreover, a stronger therapeutic alliance between patient and therapist in a 1-month MDT intervention was identified as an expected modifiable prognostic factor. Accurate prediction of annual days off work for one year and personal expenses due to LBP for one year may be difficult; however, possible contributing factors include pain self-efficacy at baseline for annual days off work for one year as well as high levels of healthful behaviors and low ÖMSQ-12-J scores at baseline and exercise adherence and reduction of disability after a month of MDT intervention for personal expenses due to LBP for one year.

## Figures and Tables

**Table 1:** Demographics and secondary outcomes of the 58 participants included in the multiple regression modeling.

Variable	Mean (SD) or Number [%]
Age (year)	45.2 (8.6)
Gender (men; women)	28 [48.3]; 30 [51.7]
Body mass index (kg/m^2^)	22.3 (3.3)
Job satisfaction (not satisfied; satisfied)	7 [12.1]; 51 [87.9]
History of recurrent low back pain (<3 times; ≥3 times)	24 [41.4]; 34 [58.6]
Job and physical activities (not physically demanding; physically demanding)	25 [43.1]; 33 [56.9]
Symptom duration (<3 months; ≥3 months)	29 [50]; 29 [50]
ÖMSQ-12-J (0–120)	52.5 (23.3)
STarT Back Screening Tool (low risk; medium-high risk)	17 [29.3]; 41 [70.7]
Five-item Modified WAI Japanese musculoskeletal version (0–5)	2.4 (2.2)
Japanese version of the Exercise Adherence Rating Scale (%)	69.8 (19.4)
MDT classification (non-derangement syndrome; derangement syndrome)	9 [15.5]; 49 [84.5]
Therapist’s MDT level (credential MDT; diploma MDT)	49 [84.5]; 9 [15.5]

Abbreviations: ÖMSQ-12-J, 12-item Örebro musculoskeletal screening questionnaire; WAI, Working Alliance Inventory-Short Form Client; MDT, Mechanical Diagnosis and Therapy.

**Table 2:** Other secondary outcomes of the 58 participants included in the multiple regression modeling.

Variable	Baseline	1-Month Follow-Up
Symptom location (0–6)	3.1 (1.5)	2.6 (1.6)
Four-item Pain Intensity Measure (0–40)	20.4 (9.6)	13.3 (9.1)
Oswestry Disability Index (%)	28.8 (16.3)	21.1 (12.2)
Central Sensitization Inventory (0–40)	23.5 (10.5)	22.4 (10.1)
Pain Self-Efficacy Questionnaire (0–60)	36.1 (14.6)	39.3 (14.3)
Tampa Scale for Kinesiophobia (17–68)	40.8 (5.5)	40.3 (4.6)
Pain Catastrophization Scale (0–52)	22.3 (11.4)	17.6 (10.6)
HeiQ–self-monitoring and insight (1–4)	2.3 (0.4)	2.5 (0.4)
HeiQ–skill and technique acquisition (1–4)	2.6 (0.4)	2.6 (0.4)
HeiQ–health-directed behavior (1–4)	2.7 (0.8)	2.8 (0.5)
HeiQ–positive and active engagement in life (1–4)	2.7 (0.5)	2.8 (0.5)
HeiQ–emotional distress (1–4)	2.4 (0.4)	2.5 (0.4)
HeiQ–constructive attitudes and approaches (1–4)	2.4 (0.5)	2.4 (0.4)
HeiQ–social integration and support (1–4)	2.2 (0.5)	2.2 (0.4)
HeiQ–health services navigation (1–4)	2.3 (0.4)	2.3 (0.4)

Values are presented with mean (SD). Abbreviations: HeiQ, Health Education Impact Questionnaire.

**Table 3:** Results of multiple regression modeling for subjective improvement after one year.

Model	Unstandardized Coefficients (B)	Standardized Coefficients (β)	*p*-Value (95% Confidence Intervals)
(Constant)	0.75		0.380 (−0.96 to 2.46)
MDT classification	1.25	0.35	<0.001 (0.54 to 1.96)
Symptom duration	1.21	0.47	<0.001 (0.66 to 1.76)
Therapeutic alliance at 1-month follow-up	0.20	0.33	0.002 (0.80 to 0.32)
STA at baseline	0.87	0.29	0.007 (0.25 to 1.48)
Pain Catastrophization Scale at baseline	−0.02	−0.21	0.034 (−0.047 to −0.002)

Abbreviations: MDT classification, whether the Mechanical Diagnosis and Therapy classification was derangement syndrome (1) or not (0); Symptom duration, whether symptom duration of this episode prior to the initial MDT session was 3 months or more (1) or less than 3 months (0); STA, skill and technique acquisition subscale of the Health Education Impact Questionnaire. *R*^2^ = 0.51, analysis of variance *p* < 0.001.

**Table 4:** Results of multiple regression modeling for annual days off work for one year.

Model	Unstandardized Coefficients (B)	Standardized Coefficients (β)	*p*-Value (95% Confidence Intervals)
(Constant)	4.25		<0.001 (2.26 to 6.23)
Pain Self-Efficacy Questionnaire at baseline	−0.06	−0.31	0.017 (−0.11 to −0.01)

Abbreviations: MDT subgroup, whether the final MDT subgroup was derangement syndrome or not. *R*^2^ = 0.10, analysis of variance *p* = 0.017.

**Table 5:** Results of multiple regression modeling for personal expenses for one year.

Model	Unstandardized Coefficients (B)	Standardized Coefficients (β)	*p*-Value (95% Confidence Intervals)
(Constant)	−35,102.99		0.67 (−201,495.63 to 131,289.66)
EARS at 1-month follow-up	2482.66	0.36	0.002 (960.75 to 4004.58)
HDB at baseline	−49,938.22	−0.29	0.014 (−89,378.33 to −10,498.11)
Reduction of ODI through 1 month	−3810.84	−0.41	0.002 (−6119.62 to −1502.05)
ÖMSQ-12-J at baseline	1738.37	0.31	0.018 (310.50 to 3166.23)

Abbreviations: EARS, Japanese version of the Exercise Adherence Rating Scale; HDB, health-directed behavior subscale of the Health Education Impact Questionnaire; ODI, Oswestry Disability Index; ÖMSQ-12-J, 12-item Örebro musculoskeletal screening questionnaire. *R*^2^ = 0.36, analysis of variance *p* < 0.001.

## Data Availability

The data presented in this study are available on request from the corresponding author. The data are not publicly available due to ethical reasons.

## References

[1] Hubertsson J., Englund M., Hallgårde U., Lidwall U., Löfvendahl S., Petersson I.F. (2014). Sick leave patterns in common musculoskeletal disorders—A study of doctor prescribed sick leave. BMC Musculoskelet. Disord..

[2] Pekkala J., Rahkonen O., Pietiläinen O., Lahelma E., Blomgren J. (2018). Sickness absence due to different musculoskeletal diagnoses by occupational class: A register-based study among 1.2 million Finnish employees. Occup. Environ. Med..

[3] March L., Smith E.U.R., Hoy D.G., Cross M.J., Sanchez-Riera L., Blyth F., Buchbinder R., Vos T., Woolf A.D. (2014). Burden of disability due to musculoskeletal (MSK) disorders. Best Pract. Res. Clin. Rheumatol..

[4] Sebbag E., Felten R., Sagez F., Sibilia J., Devilliers H., Arnaud L. (2019). The world-wide burden of musculoskeletal diseases: A systematic analysis of the World Health Organization Burden of Diseases Database. Ann. Rheum. Dis..

[5] Office for National Statistics Dataset: Sickness Absence in the UK Labour Market. https://www.ons.gov.uk/employmentandlabourmarket/peopleinwork/employmentandemployeetypes/datasets/sicknessabsenceinthelabourmarket.

[6] Gracey J.H., McDonough S.M., Baxter G.D. (2002). Physiotherapy management of low back pain: A survey of current practice in northern Ireland. Spine.

[7] Davies C., Nitz A.J., Mattacola C.G., Kitzman P., Howell D., Viele K., Baxter D., Brockopp D. (2014). Practice patterns when treating patients with low back pain: A survey of physical therapists. Physiother. Theory Pract..

[8] Foster N.E., Thompson K.A., Baxter G.D., Allen J.M. (1999). Management of nonspecific low back pain by physiotherapists in Britain and Ireland. A descriptive questionnaire of current clinical practice. Spine.

[9] Spoto M.M., Collins J. (2008). Physiotherapy diagnosis in clinical practice: A survey of orthopaedic certified specialists in the USA. Physiother. Res. Int..

[10] Machado L.A., Maher C.G., Herbert R.D., Clare H., McAuley J.H. (2010). The effectiveness of the McKenzie method in addition to first-line care for acute low back pain: A randomized controlled trial. BMC Med..

[11] de Campos T.F., Pocovi N.C., Maher C.G., Clare H.A., da Silva T.M., Hancock M.J. (2020). An individualised self-management exercise and education program did not prevent recurrence of low back pain but may reduce care seeking: A randomised trial. J. Physiother..

[12] Donelson R., Spratt K., McClellan W.S., Gray R., Miller J.M., Gatmaitan E. (2019). The cost impact of a quality-assured mechanical assessment in primary low back pain care. J. Man. Manip. Ther..

[13] Manca A., Dumville J.C., Torgerson D.J., Klaber Moffett J.A., Mooney M.P., Jackson D.A., Eaton S. (2007). Randomized trial of two physiotherapy interventions for primary care back and neck pain patients: Cost effectiveness analysis. Rheumatology.

[14] Tsuge T., Takasaki H., Toda M. (2020). Does the Keele STarT Back Screening Tool Contribute to Effectiveness in Treatment and Cost and Loss of Follow-Up of the Mechanical Diagnosis and Therapy for Patients with Low Back Pain?. Diagnostics.

[15] Takasaki H., Aoki S., May S. (2018). No increase in 6-week treatment effect of Mechanical Diagnosis and Therapy with the use of the LUMOback in people with non-acute non-specific low back pain and a directional preference of extension: A pilot randomized controlled trial. Physiotherapy.

[16] Halliday M.H., Garcia A.N., Amorim A.B., Machado G.C., Hayden J.A., Pappas E., Ferreira P.H., Hancock M.J. (2019). Treatment Effect Sizes of Mechanical Diagnosis and Therapy for Pain and Disability in Patients with Low Back Pain: A Systematic Review. J. Orthop. Sport. Phys. Ther..

[17] Kuhnow A., Kuhnow J., Ham D., Rosedale R. (2020). The McKenzie Method and its association with psychosocial outcomes in low back pain: A systematic review. Physiother. Theory Pract..

[18] Suzuki K., Takasaki H. (2020). Ability of Therapists Trained in Mechanical Diagnosis and Therapy to Guess Pain Catastrophizing and Kinesiophobia Scores for Patients with Low Back Pain. Open J. Ther. Rehabil..

[19] Takasaki H. (2017). Mechanical Diagnosis and Therapy enhances attitude towards self-management in people with musculoskeletal disorders: A preliminary evidence with a before-after design. SAGE Open Med..

[20] Supp G., Schoch W., Baumstark M.W., May S. (2020). Do patients with low back pain remember physiotherapists’ advice? A mixed-methods study on patient-therapist communication. Physiother. Res. Int..

[21] McKenzie R., May S. (2003). The Lumbar Spine: Mechanical Diagnosis and Therapy.

[22] van Helvoirt H., Apeldoorn A.T., Knol D.L., Arts M.P., Kamper S.J., van Tulder M.W., Ostelo R.W. (2016). Transforaminal epidural steroid injections influence Mechanical Diagnosis and Therapy (MDT) pain response classification in candidates for lumbar herniated disc surgery. J. Back. Musculoskelet. Rehabil..

[23] Garcia A.N., Costa L., de Souza F.S., de Almeida M.O., Araujo A.C., Hancock M., Costa L.O.P. (2018). Reliability of the Mechanical Diagnosis and Therapy System in Patients with Spinal Pain: A Systematic Review. J. Orthop. Sport. Phys. Ther..

[24] Kamper S.J., Maher C.G., Mackay G. (2009). Global rating of change scales: A review of strengths and weaknesses and considerations for design. J. Man. Manip. Ther..

[25] Lam O.T., Dumas J.P., Simon C.B., Tousignant-Laflamme Y. (2018). McKenzie mechanical syndromes coincide with biopsychosocial influences, including central sensitization: A descriptive study of individuals with chronic neck pain. J. Man. Manip. Ther..

[26] Takasaki H., May S. (2014). Mechanical diagnosis and therapy has similar effects on pain and disability as ‘wait and see’ and other approaches in people with neck pain: A systematic review. J. Physiother..

[27] Gabel P., Burkett B., Yelland M. (2009). Balancing fidelity and practicality in short version musculoskeletal patient reported outcome measures. Phys. Ther. Rev..

[28] Takasaki H., Gabel C.P. (2017). Cross-cultural adaptation of the 12-item Örebro musculoskeletal screening questionnaire to Japanese (ÖMSQ-12-J), reliability and clinicians’ impressions for practicality. J. Phys. Ther. Sci..

[29] Hoogendoorn W.E., Bongers P.M., de Vet H.C., Ariëns G.A., van Mechelen W., Bouter L.M. (2002). High physical work load and low job satisfaction increase the risk of sickness absence due to low back pain: Results of a prospective cohort study. Occup. Environ. Med..

[30] van den Heuvel S.G., Ariëns G.A., Boshuizen H.C., Hoogendoorn W.E., Bongers P.M. (2004). Prognostic factors related to recurrent low-back pain and sickness absence. Scand. J. Work Environ. Health.

[31] Stanton T.R., Latimer J., Maher C.G., Hancock M. (2009). Definitions of recurrence of an episode of low back pain: A systematic review. Spine.

[32] Machado G.C., Maher C.G., Ferreira P.H., Latimer J., Koes B.W., Steffens D., Ferreira M.L. (2017). Can Recurrence After an Acute Episode of Low Back Pain Be Predicted?. Phys. Ther..

[33] Serranheira F., Sousa-Uva M., Heranz F., Kovacs F., Sousa-Uva A. (2020). Low Back Pain (LBP), work and absenteeism. Work.

[34] Matsudaira K., Oka H., Kikuchi N., Haga Y., Sawada T., Tanaka S. (2017). The Japanese version of the STarT Back Tool predicts 6-month clinical outcomes of low back pain. J. Orthop. Sci..

[35] Miki T., Kondo Y., Takebayashi T., Takasaki H. (2020). Difference between physical therapist estimation and psychological patient-reported outcome measures in patients with low back pain. PLoS ONE.

[36] Spadoni G.F., Stratford P.W., Solomon P.E., Wishart L.R. (2004). The evaluation of change in pain intensity: A comparison of the P4 and single-item numeric pain rating scales. J. Orthop. Sport. Phys. Ther..

[37] Fairbank J.C., Couper J., Davies J.B., O’Brien J.P. (1980). The Oswestry low back pain disability questionnaire. Physiotherapy.

[38] Fujiwara A., Kobayashi N., Saiki K., Kitagawa T., Tamai K., Saotome K. (2003). Association of the Japanese Orthopaedic Association score with the Oswestry Disability Index, Roland-Morris Disability Questionnaire, and short-form 36. Spine.

[39] Tanaka K., Nishigami T., Mibu A., Manfuku M., Yono S., Shinohara Y., Tanabe A., Ono R. (2017). Validation of the Japanese version of the Central Sensitization Inventory in patients with musculoskeletal disorders. PLoS ONE.

[40] Mayer T.G., Neblett R., Cohen H., Howard K.J., Choi Y.H., Williams M.J., Perez Y., Gatchel R.J. (2012). The Development and Psychometric Validation of the Central Sensitization Inventory. Pain Pract..

[41] Adachi T., Nakae A., Maruo T., Shi K., Shibata M., Maeda L., Saitoh Y., Sasaki J. (2014). Validation of the Japanese version of the pain self-efficacy questionnaire in Japanese patients with chronic pain. Pain Med..

[42] Nicholas M.K. (2007). The pain self-efficacy questionnaire: Taking pain into account. Eur. J. Pain..

[43] Kikuchi N., Matsudaira K., Sawada T., Oka H. (2015). Psychometric properties of the Japanese version of the Tampa Scale for Kinesiophobia (TSK-J) in patients with whiplash neck injury pain and/or low back pain. J. Orthop. Sci..

[44] Miller R.P., Kori S.H., Todd D.D. (1991). The Tampa Scale: A Measure of Kinisophobia. Clin. J. Pain..

[45] Matsuoka H., Sakano Y. (2007). Assessment of Cognitive Aspect of Pain: Development, Reliability, and Validation of Japanese Version of Pain Catastrophizing Scale. Jpn. J. Psychosom. Med..

[46] Sullivan M., Bishop S.R., Pivik J. (1995). The pain catastrophizing scale: Development and validation. Psychol. Assess..

[47] Osborne R.H., Elsworth G.R., Whitfield K. (2007). The Health Education Impact Questionnaire (heiQ): An outcomes and evaluation measure for patient education and self-management interventions for people with chronic conditions. Patient Educ. Couns..

[48] Morita R., Arakida M., Osborne R.H., Nolte S., Elsworth G.R., Mikami H. (2013). Adaptation and validation of the Japanese version of the Health Education Impact Questionnaire (heiQ-J) for the evaluation of self-management education interventions. Jpn. J. Nurs. Sci..

[49] Takasaki H., Miki T., Hall T. (2018). Development of the Working Alliance Inventory-Short Form Japanese version through factor analysis and test–retest reliability. Phys. Theory Pract..

[50] Takasaki H., Kawazoe S., Miki T., Chiba H., Godfrey E. (2021). Development and validity assessment of a Japanese version of the Exercise Adherence Rating Scale in participants with musculoskeletal disorders. Health Qual. Life Outcomes.

[51] Moore D.S., Notz W., Fligner M.A. (2013). The Basic Practice of Statistics.

[52] May S., Runge N., Aina A. (2018). Centralization and directional preference: An updated systematic review with synthesis of previous evidence. Musculoskelet. Sci. Pract..

[53] Wertli M.M., Eugster R., Held U., Steurer J., Kofmehl R., Weiser S. (2014). Catastrophizing-a prognostic factor for outcome in patients with low back pain: A systematic review. Spine J..

[54] Petersen K.K., Jensen M.B., Graven-Nielsen T., Hauerslev L.V., Arendt-Nielsen L., Rathleff M.S. (2020). Pain Catastrophizing, Self-reported Disability, and Temporal Summation of Pain Predict Self-reported Pain in Low Back Pain Patients 12 Weeks After General Practitioner Consultation: A Prospective Cohort Study. Clin. J. Pain.

[55] Hall A.M., Ferreira P.H., Maher C.G., Latimer J., Ferreira M.L. (2010). The Influence of the Therapist-Patient Relationship on Treatment Outcome in Physical Rehabilitation: A Systematic Review. Phys. Ther..

[56] Kinney M., Seider J., Beaty A.F., Coughlin K., Dyal M., Clewley D. (2020). The impact of therapeutic alliance in physical therapy for chronic musculoskeletal pain: A systematic review of the literature. Phys. Theory Pract..

[57] Murray A., Hall A., Williams G.C., McDonough S.M., Ntoumanis N., Taylor I., Jackson B., Copsey B., Hurley D.A., Matthews J. (2019). Assessing physiotherapists’ communication skills for promoting patient autonomy for self-management: Reliability and validity of the communication evaluation in rehabilitation tool. Disabil. Rehabil..

[58] Handa Y., Tachikawa E., Chiba H., Kondo Y., Hamamoto T., Takasaki H. In Scores of the Communication Evaluation in Rehabilitation Tool could be dependent on physical therapists: A pilot study and secondary analysis. Proceedings of the 20th Annual Conference of the Japanese Society of Allied Health and Rehabilitation.

[59] Skagen K., Collins A.M. (2016). The consequences of sickness presenteeism on health and wellbeing over time: A systematic review. Soc. Sci. Med..

[60] Schouteten R. (2017). Predicting absenteeism: Screening for work ability or burnout. Occup. Med..

[61] Greener M.J., Guest J.F. (2005). Do antidepressants reduce the burden imposed by depression on employers?. CNS Drugs.

[62] Cancelliere C., Donovan J., Stochkendahl M.J., Biscardi M., Ammendolia C., Myburgh C., Cassidy J.D. (2016). Factors affecting return to work after injury or illness: Best evidence synthesis of systematic reviews. Chiropr. Man. Therap..

[63] Lim Y.Z., Chou L., Au R.T., Seneviwickrama K.M.D., Cicuttini F.M., Briggs A.M., Sullivan K., Urquhart D.M., Wluka A.E. (2019). People with low back pain want clear, consistent and personalised information on prognosis, treatment options and self-management strategies: A systematic review. J. Physiother..

[64] Ernstzen D.V., Hillier S.L., Louw Q.A. (2022). Synthesis of clinical practice guideline recommendations for the primary health care of chronic musculoskeletal pain. J. Eval. Clin. Pract..

[65] May S. (2007). Patients’ attitudes and beliefs about back pain and its management after physiotherapy for low back pain. Physiother. Res. Int..

[66] Slade S.C., Molloy E., Keating J.L. (2009). ‘Listen to me, tell me’: A qualitative study of partnership in care for people with non-specific chronic low back pain. Clin. Rehabil..

[67] Unsgaard-Tøndel M., Søderstrøm S. (2021). Therapeutic Alliance: Patients’ Expectations Before and Experiences After Physical Therapy for Low Back Pain-A Qualitative Study with 6-Month Follow-Up. Phys. Ther..

